# Correlation of Serum Albumin Levels With Laboratory Parameters in Automated Peritoneal Dialysis and Continuous Ambulatory Peritoneal Dialysis Patients: A Prospective Cohort Study

**DOI:** 10.7759/cureus.47364

**Published:** 2023-10-20

**Authors:** Rabab M AlMojalled, Reem M Almabadi, Ahlam A Alghamdi, Razan Z Alnugali

**Affiliations:** 1 Department of Nutrition, King Fahad Armed Forces Hospital, Jeddah, SAU; 2 Department of Nutrition and Dietetics, King Fahad Armed Forces Hospital, Jeddah, SAU; 3 Department of Health Education, King Fahad Armed Forces Hospital, Jeddah, SAU; 4 Department of Family Medicine, King Fahad Armed Forces Hospital, Jeddah, SAU

**Keywords:** automated pd, end-stage renal disease (esrd), albumin, nutrition, peritoneal dialysis (pd)

## Abstract

Background

Peritoneal dialysis (PD) is a treatment option for end-stage renal disease (ESRD) patients, with automated peritoneal dialysis (APD) and continuous ambulatory peritoneal dialysis (CAPD) being the two main modalities. APD has reported benefits such as reduced peritonitis rates, improved ultrafiltration, and enhanced quality of life. However, some studies have found potential negative consequences of APD, and the impact on survival outcomes is limited and contradictory. Selecting the appropriate PD modality for ESRD patients should be individualized based on various factors, including nutritional status, demographic factors, laboratory findings, and other outcomes. PD patients are at high risk of malnutrition, and serum albumin is commonly used as a marker of nutritional status. Continuous monitoring of laboratory values may be beneficial for identifying nutritional deficiencies in a timely manner.

Methodology

This prospective cohort study aimed to compare APD and CAPD modalities in relation to serum albumin levels, demographic factors, and other laboratory parameters. The sample consisted of patients with ESRD treated with PD, who were divided into two groups per baseline albumin level. The study collected data on demographic, clinical, and laboratory characteristics, as well as comorbidities. The data were analyzed using SPSS version 26 (IBM Corp., Armonk, NY, USA), and statistical tests, such as the chi-square test and repeated-measures analysis of variance (ANOVA), were conducted to determine significant associations and differences between variables.

Results

The study included a total of 85 patients with ESRD who required PD as a treatment modality. Among them, 71 patients were undergoing APD, and 14 patients were undergoing CAPD. The study found that there were no significant differences in demographic factors, laboratory parameters, or medical history parameters between APD and CAPD patients with different albumin levels. The patients were followed up for six months and laboratory parameters were evaluated. Repeated-measures ANOVA showed that there were no significant variations in both APD and CAPD patients. However, Spearman’s rank correlation test revealed statistically important correlations between albumin and some laboratory parameters in both APD and CAPD patients at different assessment stages, including hemoglobin, sodium, transferrin, uric acid, phosphate, total protein, cholesterol, and triglycerides (p < 0.05).

Conclusions

Serum albumin levels appeared to be unaffected by the choice of PD modality. There were significant correlations between serum albumin levels and specific laboratory findings, including total protein, across all assessment stages for both APD and CAPD patients. These findings underscore the importance of continuous laboratory monitoring for PD patients.

## Introduction

Peritoneal dialysis (PD) is an effective and convenient treatment for end-stage renal disease (ESRD) patients who are unable or unwilling to undergo hemodialysis [1]. The treatment procedure entails the exchange of fluid and solutes through the peritoneal membrane, which facilitates the effective removal of waste products. Continuous ambulatory peritoneal dialysis (CAPD) is a form of PD in which the patient manually performs the PD exchanges. On the other hand, automated peritoneal dialysis (APD) is a general term used to describe all types of PD that use a mechanical device to facilitate the delivery and removal of dialysate. There are several types of APD, including continuous cyclical PD, intermittent PD, nightly intermittent PD, and tidal PD. Unlike APD, CAPD requires the patient or caregiver to perform a minimum of four to five exchanges each day [2].

Numerous studies have reported significant advantages of APD compared to CAPD, such as reduced peritonitis rates, improved ultrafiltration, enhanced quality of life, increased small solute clearances, and decreased mechanical complications [1,3]. However, not all investigations have confirmed these benefits, and some authors have observed potential negative consequences of APD, such as increased cost and accelerated loss of residual renal function [4,5]. The impact of PD modality on survival outcomes is also limited and contradictory. Some studies have reported improved overall survival and technique success rates in patients treated with APD compared to those treated with CAPD, while others have found no differences in outcomes between the two modalities [6,7]. However, many of these investigations are limited by potential biases and inadequate statistical adjustment for differences in baseline characteristics between the APD and CAPD populations.

The utilization of PD has been linked to several enduring pathophysiological alterations that lead to negative health outcomes, including malnutrition, which affects 30-50% of patients [8]. Malnutrition can complicate PD treatment by contributing to the development of infections, while infectious diseases can, in turn, directly impact nutritional intake and adequacy. Top-tier guidelines for PD advise on the importance of regularly and comprehensively assessing patient appetite, measuring body weight, monitoring clinical status, evaluating dietary intake, and measuring laboratory markers of nutrition, including albumin, potassium, bicarbonate, and phosphate [9].

There are several established methods for stratifying malnutrition in PD, with one commonly used marker being serum albumin. The utility of albumin as a marker of nutritional status stems from its sensitivity to changes in dietary protein intake. Additionally, albumin is inexpensive and readily available [10]. However, its diagnostic value is limited as albumin levels may be influenced by various factors, including liver disease, gastrointestinal and renal losses, and inflammatory states characterized by high levels of tumor necrosis factor-alpha (TNF-α) and interleukin 6 (IL-6). As a result, albumin levels are primarily used as a screening tool in clinical practice [11].

Selecting the appropriate PD modality for ESRD patients should be individualized based on a variety of factors, including nutritional status, demographic factors, laboratory findings, and other outcomes. A comprehensive understanding of the differences between CAPD and APD can assist healthcare providers in making informed decisions and optimizing patient outcomes. In this study, we aim to compare APD and CAPD in correlation with baseline nutritional status, demographic factors, and laboratory findings. This may help clinicians make more informed treatment decisions and encourage them to monitor the nutritional status of patients using a variety of lab parameters.

## Materials and methods

Study design

We conducted a prospective cohort study comparing the characteristics of patients undergoing CAPD and APD in correlation with serum albumin levels. We enrolled patients with ESRD who required PD at the Renal Disease and Transplant Center in King Fahad Armed Forces Hospital from October 2020 to March 2022. Patients were grouped according to their PD modality (APD and CAPD) and baseline serum albumin level. The cut-off for serum albumin was set at 35 g/L, and patients were divided into the following two groups group I with albumin levels equal to or above the cut-off value, and group II with albumin levels less than 35 g/L. Other factors assessed included demographic (age, gender), clinical (weight, height, body mass index), and laboratory (glucose, blood urea nitrogen (BUN), creatinine, hemoglobin, sodium, potassium, phosphate, C-reactive protein (CRP), cholesterol, triglycerides) characteristics. We also included data on comorbidities. Lab parameters were collected monthly for six months. The abovementioned factors were compared between group I and group II and PD modalities.

Study participants

The sample consisted of 85 patients with ESRD treated at the Renal Disease and Transplant Center in King Fahad Armed Forces Hospital. Patients ≥18 years old with ESRD diagnosed according to Kidney Disease Improving Global Outcomes diagnostic criteria and PD as a chosen treatment modality were included in the study while patients with missing or incomplete medical records were excluded. The APD group comprised 71 patients, and the CAPD group included 14 patients. According to baseline serum albumin level, 31 patients were allocated to group I and 54 to group II.

Data and sample collection

For the assessment of laboratory indicators, a comprehensive metabolic panel was taken monthly. Blood samples were drawn at the hospital pre-meal and were later analyzed in the hospital laboratory. Patient data including medical history and laboratory results were extracted from electronic medical records and meticulously organized into Excel spreadsheets. This prospective cohort study complied with the Declaration of Helsinki guidelines and was approved by the ethics committee of King Fahad Armed Forces Hospital, Jeddah (reference number: REC 498).

Statistical analysis

Data were entered into Microsoft Excel (Microsoft Corp., Redmond, WA, USA) and analyzed using SPSS version 26 (IBM Corp., Armonk, NY, USA). Means and standard deviations (SDs) were used to present continuous data, while frequencies and percentages were used to present categorical data. The multiple imputation method was applied to replace missing values in the dataset. Analysis was conducted on both APD and CAPD patients separately and compared with albumin levels groups. Patients were classified into two groups according to their albumin level, which is a good marker of nutritional condition. The chi-square test was performed to determine significant associations between albumin levels and medical history parameters, and a p-value <0.05 was considered significant. A repeated-measures analysis of variance (ANOVA) was conducted to examine the differences in means of laboratory parameters at different time points. Additionally, Spearman’s rank correlation test was performed to investigate the linear relationship between albumin and other laboratory parameters.

## Results

The present study evaluated a cohort consisting of 71 APD patients and 14 CAPD patients. Demographically, a male predominance was observed in the APD group (56.34%), while females were more prevalent in the CAPD group (57.14%) (Table 1). In terms of age, the APD cohort displayed a mean age of 48.21 ± 17.42 years, whereas the CAPD cohort had a younger mean age of 40.30 ± 14.39 years (Table 1). When examining anthropometric parameters, the APD patients had a mean weight of 67.89 ± 15.83 kg and a mean height of 160.79 ± 9.07 cm. For the CAPD patients, the corresponding values were 63.28 ± 21.04 kg and 155.77 ± 11.51 cm (Table 1). The study also evaluated body mass index (BMI) across both patient groups. The APD patients exhibited a mean BMI of 26.07 ± 5.07 kg/m^2^, closely followed by the CAPD patients with a mean BMI of 25.70 ± 6.40 kg/m^2^ (Table 1).

**Table 1:** Demographic characteristics of the patients. APD: automated peritoneal dialysis; CAPD: continuous ambulatory peritoneal dialysis; SD: standard deviation; BMI: body mass index; n: numbers; %: percentage

	APD (mean ± SD)	CAPD (mean ± SD)
Age	48.21 ± 17.42	40.30 ± 14.39
Weight	67.89 ± 15.83	63.28 ± 21.04
Height	160.79 ± 9.07	155.77 ± 11.51
BMI	26.07 ± 5.07	25.70 ± 6.40
Gender	n (%)	n (%)
Female	31 (43.66%)	8 (57.14%)
Male	40 (56.34%)	6 (42.86%)

Repeated-measures ANOVA for lab parameters at different months

A longitudinal analysis was performed on various laboratory parameters among APD patients across a six-month period, employing repeated-measures ANOVA with Greenhouse-Geisser correction for statistical rigor. The variables assessed included glucose, BUN, creatinine, hemoglobin, transferrin, sodium, potassium, albumin, uric acid, phosphate, CRP, total protein, cholesterol, and triglycerides. Our analyses did not reveal any statistically significant changes in the majority of the laboratory parameters over the follow-up period. P-values were all above the 0.05 threshold, indicating a lack of statistically significant variations across the different time points (Table 2).

**Table 2:** Descriptive statistics for all lab parameters: APD. BUN: blood urea nitrogen; Na: sodium; K: potassium; CRP: C-reactive protein; SD: standard deviation; BMI: body mass index

	Assessment stage						
Variables	First month	Second month	Third month	Fourth month	Fifth month	Sixth month	P-value
	Mean ± SD	Mean ± SD	Mean ± SD	Mean ± SD	Mean ± SD	Mean ± SD	
Glucose (mg/dL)	8.53 ± 5.79	8.27 ± 3.77	8.38 ± 2.51	8.09 ± 3.42	8.29 ± 3.53	7.71 ± 2.74	0.70
BUN (mg/dL)	17.68 ± 5.42	16.92 ± 5.19	16.53 ± 6.18	16.41 ± 5.79	15.90 ± 4.74	16.71 ± 4.54	0.28
Creatinine (mg/dL)	745.38 ± 270.34	696.35 ± 307.99	701.88 ± 271.70	741.27 ± 262.60	774.74 ± 300.40	764.13 ± 259.57	0.15
Hemoglobin (g/L)	108.62 ± 15.30	105.07 ± 14.71	107.19 ± 14.04	105.22 ± 15.03	104.11 ± 16.45	102.62 ± 16.05	0.05
Transferrin (mg/dL)	1.86 ± 0.24	1.82 ± 0.27	1.85 ± 0.38	1.81 ± 0.24	1.74 ± 0.25	1.79 ± 0.21	0.06
Na (mmol/L)	134.56 ± 2.95	134.44 ± 3.58	135.44 ± 5.73	134.48 ± 3.81	134.66 ± 3.33	134.69 ± 3.31	0.50
K (mmol/L)	4.21 ± 0.43	4.18 ± 0.51	4.23 ± 0.55	4.22 ± 0.66	4.08 ± 0.51	4.11 ± 0.58	0.34
Albumin (g/dL)	34.08 ± 4.47	34.31 ± 3.81	38.07 ± 31.65	33.60 ± 3.63	33.93 ± 6.06	33.80 ± 4.24	0.30
Uric acid (mg/dL)	397.65 ± 76.80	396.46 ± 64.30	411.92 ± 123.45	378.54 ± 77.20	396.79 ± 91.30	384.92 ± 76.93	0.19
Phosphate (mg/dL)	1.72 ± 0.40	1.76 ± 0.39	1.69 ± 0.43	1.65 ± 0.45	1.70 ± 0.47	1.70 ± 0.43	0.57
CRP (mg/dL)	24.61 ± 27.86	22.24 ± 17.21	16.28 ± 39.91	24.63 ± 24.60	26.80 ± 38.26	21.27 ± 18.33	0.32
Total protein (g/dL)	66.23 ± 5.80	65.41 ± 4.32	67.59 ± 15.43	66.42 ± 8.66	65.89 ± 5.35	65.58 ± 6.40	0.55
Cholesterol (mg/dL)	4.60 ± 0.85	4.61 ± 0.64	4.77 ± 0.71	4.48 ± 0.63	4.60 ± 0.98	4.60 ± 0.49	0.20
Triglycerides (mg/dL)	1.74 ± 0.64	1.69 ± 0.53	1.80 ± 0.73	1.73 ± 0.55	1.67 ± 0.61	1.67 ± 0.46	0.65

Similarly, a comprehensive, longitudinal analysis was performed to evaluate the temporal changes in various biochemical and hematological parameters among the study cohort over a period of six months. The analysis demonstrated that none of the parameters exhibited statistically significant changes across the six-month period. All parameters displayed p-values exceeding the conventional 0.05 threshold for statistical significance. This suggests a relative stability in these markers over the six-month assessment period (Table 3). Statistical comparison between these groups could not be performed due to unequal population distribution among groups.

**Table 3:** Descriptive statistics for all the lab parameters: CAPD. BUN: Blood urea nitrogen; Na: sodium; K: potassium; CRP: C-reactive protein; SD: standard deviation; BMI: body mass index

	Assessment stage						
Variables	First month	Second month	Third month	Fourth month	Fifth month	Sixth month	P-value
	Mean ± SD	Mean ± SD	Mean ± SD	Mean ± SD	Mean ± SD	Mean ± SD	
Glucose (mg/dL)	5.62 ± 0.45	5.64 ± 0.75	5.80 ± 0.80	5.23 ± 1.18	5.71 ± 1.34	5.41 ± 1.00	0.55
BUN (mg/dL)	16.03 ± 2.83	14.54 ± 5.20	14.67 ± 5.25	15.44 ± 4.89	16.70 ± 4.96	16.70 ± 4.96	0.80
Creatinine (mg/dL)	750.94 ± 210.31	720.86 ± 350.38	668.61 ± 235.80	736.39 ± 346.88	661.51 ± 361.15	666.76 ± 273.05	0.80
Hgb (g/L)	105.03 ± 5.18	107.77 ± 8.43	108.94 ± 17.57	102.30 ± 13.33	99.22 ± 15.64	100.15 ± 15.92	0.12
Transferrin (mg/dL)	1.725 ± 0.103	1.734 ± 0.267	1.725 ± 0.204	1.678 ± 0.252	1.652 ± 0.234	1.75 ± 0.31	0.81
Na (mmol/L)	134.71 ± 2.56	134.43 ± 3.86	135.86 ± 3.16	136.21 ± 3.56	134.86 ± 3.57	134.57 ± 3.44	0.41
K (mmol/L)	4.248 ± 0.514	4.186 ± 0.361	4.02 ± 0.26	4.02 ± 0.40	4.08 ± 0.24	4.18 ± 0.56	0.42
Albumin (g/dL)	36.15 ± 1.64	36.09 ± 2.53	36.34 ± 3.12	35.47 ± 3.56	36.23 ± 2.76	35.19 ± 4.50	0.82
Uric acid (mg/dL)	403.21 ± 38.74	404.14 ± 103.96	433.14 ± 77.61	405.36 ± 63.54	414.86 ± 55.49	400.21 ± 55.27	0.48
Phosphate (mg/dL)	1.73 ± 0.31	1.67 ± 0.43	1.68 ± 0.42	1.60 ± 0.48	1.58 ± 0.32	1.50 ± 0.39	0.46
CRP (mg/dL)	12.50 ± 3.82	13.43 ± 7.93	13.50 ± 9.07	12.50 ± 7.61	11.00 ± 7.52	15.00 ± 14.85	0.75
Total protein (g/dL)	69.14 ± 2.45	69.36 ± 4.67	69.93 ± 4.67	70.57 ± 6.15	68.00 ± 5.91	67.50 ± 7.33	0.49
Cholesterol (mg/dL)	3.49 ± 0.87	3.76 ± 1.39	3.74 ± 0.80	3.49 ± 1.32	3.40 ± 1.05	3.28 ± 0.71	0.63
Triglycerides (mg/dL)	1.35 ± 0.25	1.37 ± 0.56	1.26 ± 0.41	1.26 ± 0.53	1.29 ± 0.09	1.15 ± 0.43	0.68

Association between baseline nutritional status and the medical history of patients

Table 4 explores the prevalence of various health conditions among APD and CAPD patients based on their albumin levels, either normal (≥35 g/L) or low (≤35 g/L).

**Table 4:** Chi-square test of association. APD: automated peritoneal dialysis; CAPD: continuous ambulatory peritoneal dialysis; N: number

Variables		APD			CAPD		
		Normal albumin level (≥35 g/L)	Low albumin level (≤35 g/L)	P-value	Normal albumin level (≥35 g/L)	Low albumin level (≤35 g/L)	P-value
		N (%)	N (%)		N (%)	N (%)	
Diabetic	No	17 (60.71)	22 (51.16)	0.43	10 (76.90)	1 (100.00)	0.77
	Yes	11 (39.29)	21 (48.84)		3 (23.10)	0 (0.00)	
Hypertension	No	2 (7.14)	6 (13.95)	0.47	3 (23.10)	0 (0.00)	0.77
	Yes	26 (92.86)	37 (6.98)		10 (76.90)	1 (100.00)	
Dyslipidemia	No	26 (92.86)	38 (88.37)	0.70	12 (92.30)	1 (100.00)	0.93
	Yes	2 (7.14)	5 (11.63)		1 (7.70)	0 (0.00)	
Heart disease	No	25 (89.29)	35 (81.40)	0.51	10 (76.90)	1 (100.00)	0.79
	Yes	3 (10.71)	8 (18.60)		3 (23.10)	0 (0.00)	
Hypothyroidism	No	27(96.43)	41 (95.35)	1.00	13 (100.00)	1 (100.00)	NA
	Yes	1 (3.57)	2 (4.65)		0 (0.00)	0 (0.00)	
Hepatitis	No	26 (92.86)	41 (95.35)	0.64	12 (92.30)	1 (100.00)	0.93
	Yes	2 (7.14)	2 (4.65)		1 (7.70)	0 (0.00)	

Regarding the APD group, among patients with normal albumin levels, 60.71% (N = 17) had no history of diabetes compared to 39.29% (N = 11) who were diabetic. In contrast, the low albumin group showed a higher percentage of diabetics (48.84%, N = 21) compared to non-diabetics (51.16%, N = 22). The p-value of 0.43 indicates no statistical significance between the two groups based on diabetic status. Moreover, the prevalence of hypertension was notably higher among patients with both normal and low albumin levels (92.86%, N = 26 and 86.05%, N = 37, respectively), with a p-value of 0.47. Similar trends were observed for dyslipidemia and heart disease, with p-values of 0.70 and 0.51, respectively, indicating no statistically significant differences based on albumin levels (Table 4).

On the other hand, in the CAPD group, among those with diabetes, 76.9% (N = 10) had no history in the normal albumin group, while 100% (N = 1) in the low albumin level group had no history, with a p-value of 0.77. A similar distribution was observed for hypertension, dyslipidemia, and heart disease, with p-values of 0.77, 0.93, and 0.79, respectively, suggesting no statistically significant differences based on albumin levels (Table 4).

For both hypothyroidism and hepatitis, the percentages remained relatively consistent across albumin levels in both the APD and CAPD groups, with p-values indicating no statistical significance. In summary, various health conditions such as diabetes, hypertension, dyslipidemia, heart disease, hypothyroidism, and hepatitis did not show a statistically significant correlation with albumin levels in both APD and CAPD groups.

Relationship between albumin level and different lab parameters at different time points

Table 5 reports Spearman’s rank correlation (ρ) values and the corresponding p-values to assess the relationship between albumin levels and various other clinical variables across six months in APD patients.

**Table 5:** Correlation results of albumin with lab parameters at different time points: APD. ^#^: Fairly strong - positive correlation; ^ᶿ^: Moderate positive - correlation; ⸊: fairly strong negative - correlation; ^©^: moderate negative - correlation; ^€^: weak positive - correlation; ^µ^: weak negative correlation. *: Significant (p-value <0.05). APD: automated peritoneal dialysis; BUN: blood urea nitrogen; Na: sodium; K: potassium; CRP: C-reactive protein

Spearman’s rank correlation (ρ)													
First month (baseline)													
Variables	Glucose	BUN	Creatinine	Hemoglobin	Transferrin	Na	K	Uric acid	Phosphate	CRP	Total protein	Cholesterol	Triglycerides
Albumin	-0.05	0.08	0.19	0.38ᶿ	0.33ᶿ	0.30ᶿ	0.07	1.58	0.10	0.25ᶿ	0.75^#^	-0.03	0.07
P-value	0.70	0.50	0.11	0.00*	0.02*	0.01*	0.54	1.19	0.20	0.03*	0.00*	0.78	0.56
Second month													
Variables	Glucose	BUN	Creatinine	Hemoglobin	Transferrin	Na	K	Uric acid	Phosphate	CRP	Total protein	Cholesterol	Triglycerides
Albumin	0.20	0.49ᶿ	0.19	0.13	0.40ᶿ	0.35ᶿ	0.18	0.30ᶿ	0.31ᶿ	-0.17	0.60ᶿ	-0.13	0.17
P-value	0.10	0.00*	0.11	0.30	0.00*	0.00*	0.14	0.01*	0.00*	0.16	0.00*	0.26	0.17
Third month													
Variables	Glucose	BUN	Creatinine	Hemoglobin	Transferrin	Na	K	Uric acid	Phosphate	CRP	Total protein	Cholesterol	Triglycerides
Albumin	-0.16	0.06	0.10	0.25^€^	0.40ᶿ	0.27^€^	0.09	0.22	0.08	-0.00	0.68ᶿ	0.10	0.18
P-value	0.19	0.65	0.40	0.04*	0.00*	0.02*	0.48	0.07	0.54	0.10	0.00*	0.42	0.13
Fourth month													
Variables	Glucose	BUN	Creatinine	Hemoglobin	Transferrin	Na	K	Uric acid	Phosphate	CRP	Total protein	Cholesterol	Triglycerides
Albumin	-0.00	-0.00	0.15	0.22	0.29^€^	0.23	0.17	0.29^€^	0.28^€^	-0.22	0.34ᶿ	0.25^€^	-0.19
P-value	0.98	0.99	0.22	0.06	0.01*	0.05	0.17	0.01*	0.01*	0.06	0.00*	0.03*	0.10
Fifth month													
Variables	Glucose	BUN	Creatinine	Hemoglobin	Transferrin	Na	K	Uric acid	Phosphate	CRP	Total protein	Cholesterol	Triglycerides
Albumin	0.15	0.17	0.08	0.19	0.33^€^	0.34^€^	0.16	0.47ᶿ	0.23	-0.36 ^µ^	0.57ᶿ	0.22^€^	0.24
P-value	0.22	0.17	0.52	0.12	0.00	0.00	0.20	0.00*	0.05	0.00*	0.00*	0.00*	0.06
Sixth month													
Variables	Glucose	BUN	Creatinine	Hemoglobin	Transferrin	Na	K	Uric acid	Phosphate	CRP	Total protein	Cholesterol	Triglycerides
Albumin	0.02	0.06	0.14	0.17	0.20	0.21	0.15	0.39ᶿ	0.18	-0.08	0.45ᶿ	0.05	0.25^€^
P-value	0.89	0.61	0.25	0.16	0.08	0.07	0.20	0.00*	0.12	0.52	0.00*	0.70	0.03*

For the first month, a significant positive correlation was observed between albumin and hemoglobin (ρ = 0.38, p = 0.00), transferrin (ρ = 0.33, p = 0.02), sodium (ρ = 0.30, p = 0.01), and total protein (ρ = 0.75, p = 0.00). For the second month, strong correlations were noticed between albumin and BUN (ρ = 0.49, p = 0.00), transferrin (ρ = 0.40, p = 0.00), sodium (ρ = 0.35, p = 0.00), uric acid (ρ = 0.30, p = 0.01), phosphate (ρ = 0.31, p = 0.00), and total protein (ρ = 0.60, p = 0.00). Regarding the third month, albumin showed significant correlations with hemoglobin (ρ = 0.25, p = 0.04), transferrin (ρ = 0.40, p = 0.00), sodium (ρ = 0.27, p = 0.02), and total protein (ρ = 0.68, p = 0.00). Meanwhile, the fourth month showed significant correlations for transferrin (ρ = 0.29, p = 0.01), sodium (ρ = 0.23, p = 0.05), uric acid (ρ = 0.29, p = 0.01), phosphate (ρ = 0.28, p = 0.01), total protein (ρ = 0.34, p = 0.00), and cholesterol (ρ = 0.25, p = 0.03). Moreover, in the fifth month, albumin demonstrated significant relationships with transferrin (p = 0.00), sodium (p = 0.00), uric acid (p = 0.00), CRP (p = 0.00), total protein (p = 0.00), and cholesterol (p = 0.00). Lastly, the sixth month demonstrated significant positive correlations between albumin and uric acid (ρ = 0.39, p = 0.00), total protein (ρ = 0.45, p = 0.00), and triglycerides (ρ = 0.25, p = 0.03) (Table 5).

In summary, albumin levels showed various degrees of significant correlations with multiple clinical parameters across the six months. Notably, total protein remained consistently and significantly correlated with albumin levels throughout the study period

Table 6 reports Spearman’s rank correlation (ρ) values and corresponding p-values to assess the relationship between albumin levels and various other clinical variables across six months in CAPD patients. In the first month, glucose showed a moderate and statistically significant positive correlation with albumin (p = 0.02). In addition, transferrin showed a strong and statistically significant positive correlation with albumin (p = 0.00). However, all other parameters were statistically insignificant. During the second month, there were no significant correlations between albumin and most of the variables, except for phosphate, which showed a strong negative correlation that was statistically significant (p = 0.01). In the third month, the most notable finding was a strong and statistically significant positive correlation between potassium and albumin (p = 0.00). For the fourth month, albumin showed a strong and statistically significant negative correlation with CRP (p = 0.00) and a strong positive correlation with total protein which was also statistically significant (p = 0.00). In the fifth month, albumin and total protein continued to be strongly and significantly correlated (p = 0.00). Phosphate showed a moderate positive correlation that was statistically significant (p = 0.05). No other variables showed statistically significant correlations with albumin. In the sixth month, no variables showed statistically significant correlations with albumin (Table 6).

**Table 6:** Correlation results of albumin with lab parameters at different time points: CAPD. ^#^: Fairly strong positive correlation; ᶿ: moderate positive correlation; ⸊: fairly strong negative correlation; ^©^: moderate negative correlation; ^¥^: very strong positive correlation. *: Significant (p-value <0.05). BUN: blood urea nitrogen; CAPD: continuous ambulatory peritoneal dialysis; Na: sodium; K: potassium; CRP: C-reactive protein

Spearman’s rank correlation (ρ)													
First month													
Variables	Glucose	BUN	Creatinine	Hemoglobin	Transferrin	Na	K	Uric acid	Phosphate	CRP	Total protein	Cholesterol	Triglycerides
Albumin	0.61^#^	-0.23	-0.11	0.14	0.99^¥^	0.62^#^	0.32	0.09	0.57ᶿ	0.15	-0.09	0.09	-0.15
P-value	0.02*	0.44	0.71	0.64	0.00*	0.02*	0.27	0.77	0.03*	0.61	0.77	0.75	0.61
Second month													
Variables	Glucose	BUN	Creatinine	Hemoglobin	Transferrin	Na	K	Uric acid	Phosphate	CRP	Total protein	Cholesterol	Triglycerides
Albumin	0.18	-0.18	-0.25	0.33	0.14	0.45	-0.25	0.13	-0.64⸊	-0.48	0.39	-0.19	0.24
P-value	0.54	0.54	0.39	0.25	0.63	0.11	0.40	0.66	0.01*	0.08	0.17	0.52	0.41
Third month													
Variables	Glucose	BUN	Creatinine	Hemoglobin	Transferrin	Na	K	Uric acid	Phosphate	CRP	Total protein	Cholesterol	Triglycerides
Albumin	0.34	-0.40	-0.39	0.09	0.03	-0.05	0.77^#^	0.30	0.10	-0.52	0.44	0.04	-0.02
P-value	0.23	0.15	0.17	0.77	0.92	0.87	0.00*	0.30	0.74	0.06	0.11	0.90	0.96
Fourth month													
Variables	Glucose	BUN	Creatinine	Hemoglobin	Transferrin	Na	K	Uric acid	Phosphate	CRP	Total protein	Cholesterol	Triglycerides
Albumin	0.08	-0.57^©^	-0.07	0.60#	0.44	0.03	0.43	-0.09	-0.21	-0.79⸊	0.71^#^	0.00	-0.40
P-value	0.79	0.03*	0.82	0.02*	0.11	0.93	0.13	0.77	0.46	0.00*	0.00*	0.99	0.15
Fifth month													
Variables	Glucose	BUN	Creatinine	Hemoglobin	Transferrin	Na	K	Uric acid	Phosphate	CRP	Total protein	Cholesterol	Triglycerides
Albumin	0.27	-0.24	-0.31	0.08	-0.17	0.26	-0.07	0.19	0.54	-0.27	0.72^#^	-0.58^©^	0.12
P-value	0.35	0.42	0.28	0.79	0.57	0.38	0.82	0.52	0.05	0.36	0.00*	0.03*	0.69
Sixth month													
Variables	Glucose	BUN	Creatinine	Hemoglobin	Transferrin	Na	K	Uric acid	Phosphate	CRP	Total protein	Cholesterol	Triglycerides
Albumin	0.24	-0.42	-0.43	-0.27	0.21	0.01	0.19	0.29	0.06	-0.28	0.26	-0.49	-0.35
P-value	0.42	0.14	0.12	0.35	0.48	0.97	0.52	0.31	0.83	0.34	0.37	0.08	0.23

Throughout the six months, total protein remained consistently correlated with albumin, albeit not always reaching statistical significance. Variables such as transferrin, potassium, and CRP showed significant correlations in specific months.

## Discussion

The study aimed to determine whether there were significant differences in the mean values of various laboratory parameters over time in APD and CAPD patients. The results showed that there were no statistically significant differences in any of the lab parameters, including BUN, creatinine, hemoglobin, transferrin, albumin, uric acid, phosphate, total protein, cholesterol, and triglycerides, across the follow-up months. Similarly, in the study by Guney et al., no statistically significant difference was observed between the APD and CAPD groups. Except for serum creatinine, phosphorus, and calcium-phosphate product, the two groups did not significantly differ in terms of serum urea, calcium, intact parathyroid hormone, albumin, hemoglobin, CRP, total cholesterol, triglycerides, and urea clearance index (Kt/V). APD patients had significantly greater serum creatinine, phosphorus, and calcium-phosphate product levels than CAPD patients. In comparison to ADP patients, mean residual urine outputs tended to be higher in CAPD patients [12]. Previous research suggests that protein and amino acid losses are similar in APD and CAPD patients; however, the exact role of this element in the overall malnutrition of PD patients is still debatable. In PD patients, peritoneal protein loss is a significant negative predictor of albuminemia [13].

While PD is an effective and convenient treatment for ESRD patients who are unable or unwilling to undergo hemodialysis, there is a debate regarding the most appropriate modality to use. APD is often cited as having significant advantages over CAPD, such as reduced peritonitis rates, improved ultrafiltration, enhanced quality of life, increased small solute clearances, and decreased mechanical complications [1]. However, some studies have found potential negative consequences of APD, such as increased cost and accelerated loss of residual renal function, leading to contradicting results [4]. Presently, the selection of appropriate PD modality for ESRD patients is individualized or tailored according to certain factors, including nutritional status, demographic factors, laboratory findings, and other outcomes. Previous research has shown that individuals undergoing PD have notably lower levels of serum albumin compared to others [14]. Lower levels of albumin have been linked with increased mortality in these patients [15]. Although numerous factors can affect albumin levels, serum albumin is frequently used to evaluate protein malnutrition. Previous studies have reported reduced serum albumin levels in PD patients compared to those undergoing hemodialysis. This is primarily due to daily peritoneal protein losses in the dialysate effluent, which mainly consists of albumin [16].

Findings of a systematic review demonstrated a strong association between the onset of peritonitis and a low serum albumin level at the onset of CAPD. As a result, hypoalbuminemia can be used to detect peritonitis in CAPD patients. Hence, to avoid peritonitis, prompt action is needed when the serum albumin level drops [17]. Similarly, Ma et al. concluded that early-onset peritonitis is substantially linked with a higher Charlson Comorbidity Index score, a lower albumin level, and a lower Kt/V at the start of the PD. A significant peritonitis rate and a worse clinical outcome are also predicted by early-onset peritonitis [18]. In CAPD patients, the development of hypoalbuminemia is multifactorial. Important factors that affect hypoalbuminemia include advanced age, the origin of renal failure, transport status, chronic inflammation, the presence of nephrotic syndrome, and nutritional status [19].

Furthermore, the results of this study demonstrated significant correlations between albumin and some laboratory parameters at different assessment stages in both APD and CAPD patients. In APD patients, these correlations included moderate positive correlations between albumin and hemoglobin, sodium, transferrin, and total protein at different assessment stages and a weak positive correlation between albumin and triglycerides at the sixth month of observation. In CAPD patients, the correlations included very strong positive correlations between albumin and transferrin in the first month, moderate positive correlations between albumin and potassium and total protein in the third and fourth months, respectively, and a fairly strong negative correlation between albumin and phosphate in the second month. The fact that the albumin synthesis rate increases in response to extracorporeal albumin loss in CAPD patients [20] aligns with the very strong positive correlation between albumin and transferrin observed in the first month. This correlation may indeed reflect the body’s compensatory response to maintain adequate albumin levels in the face of peritoneal albumin loss during CAPD. The variation in daily transperitoneal albumin loss mentioned in the studies ranging from 2.7 to 6.6 g [21] could explain the fluctuating correlations observed over different time periods in our study, as these losses might influence albumin levels and their correlation with other markers. Additionally, the studies’ observation that some CAPD patients maintain stable serum albumin levels despite higher transperitoneal loss in the absence of inflammation or malnutrition [22] suggests that factors beyond nutritional status, such as individual patient physiology and treatment response, may also contribute to correlations observed in our study.

However, Gunalay et al. reported that levels of albumin, creatinine, low-density lipoprotein, hemoglobin, calcium, potassium, and phosphorus were not significantly different among PD patients in the various malnutrition categories [20]. Findings of a study by Kim et al demonstrated that when compared to individuals without hypokalemia, serum albumin, calcium-phosphate product, triglycerides, BMI, protein nitrogen appearance, and lean body mass were all significantly lower. Patients with hypokalemia had significantly greater serum CRP. The ultrafiltration volume at the peritoneal equilibration test and the blood albumin level were found to be independent risk variables for hypokalemia by multivariate stepwise linear regression analysis. This shows that in CAPD patients, the serum potassium level may serve as a key nutritional signal [21].

Several nutritional factors have become effective predictors of mortality in dialysis patients over the past 10 years. In hemodialysis and PD patients, measurements of serum albumin, cholesterol, protein, and creatinine reserves have been well-described predictors of survival. Among the more recent measures, serum albumin has drawn more and more attention. Albumin is a very sensitive indicator of nutritional status because of its high tryptophan concentration, short half-life, rapid turnover rate, and small pool size [22]. The substantial mortality-predictability of various markers, including hypoalbuminemia, low serum cholesterol levels, a low BMI, and decreased dietary protein intake, has been consistently demonstrated in recent research on chronic renal disease, including populations receiving dialysis. Low serum albumin is one of many nutritional markers that is a reliable indicator of death in dialysis patients. However, serum albumin has a 20-day-long half-life and is susceptible to a variety of conditions, including inflammation, patient fluid status, and losses to dialysate. Prealbumin, on the other hand, has a half-life that is two days shorter and can be a more accurate biomarker of a patient’s nutritional state. Serum BUN and creatinine are indicators of reserved renal function and have a significant impact on the nutritional status of dialysis patients. Patient nutrition status deteriorates when reserved renal function decreases. The nutritional and calorie losses during dialysis put CAPD patients at risk for protein energy malnutrition. However, several nutritional factors, such as serum pre-albumin, Subjective Global Assessment grade, and lipid profiles, were better in CAPD patients than in hemodialysis patients. It suggests that individuals receiving hemodialysis for malnutrition may require a more intense therapeutic strategy [23].

Malnutrition further causes complications in PD as evidence from the current research shows that malnourished ESRD patients experience increased risks of infection, poor rehabilitation, and mortality in addition to other issues. Such issues may be strongly related to protein-energy waste, which is the best indicator of mortality in CKD patients and is frequently observed. Hence, proper nutrition is vital for PD to be successful [9]. Gelder et al. reported that hypoalbuminemia is a potent predictor of cardiovascular disease and all-cause mortality in dialysis patients. The causes of hypoalbuminemia, such as malnutrition and inflammation, which each have a negative impact on outcomes, can be linked to the risk associated with hypoalbuminemia [24]. Heimburger et al. suggested that patients with CAPD who are underweight and hypoalbuminemic should consume more protein; if this does not work, the dosage of their dialysis should be raised. Additionally, the use of PD solutions based on amino acids is a potential novel technique for the treatment of malnourished CAPD patients and could eventually play a significant role in CAPD therapy. On the other hand, the patient should preferably be transferred to hemodialysis if the nutritional status continues to deteriorate despite these attempts [25].

APD often employs glucose-based dialysate solutions, which can lead to increased glucose absorption. This may result in hyperglycemia in some patients, potentially exacerbating diabetes if present. CAPD, on the other hand, may have more stable glucose levels as the dialysate is typically dextrose-free. Clinicians must carefully monitor glucose levels and adjust dialysate solutions accordingly to prevent metabolic complications in APD patients [26,27]. This explains increased glucose levels in APD patients than CAPD patients in our study. Williams et al. reported quite an interesting finding as they observed that compared to CAPD, APD was linked to a considerably higher nocturnal glucose level. In contrast to controls and CAPD patients, there was no discernible decrease in nocturnal glucose in APD patients relative to the daytime norm [28]. Overall, the study suggests that the lab parameters remained stable over time in both APD and CAPD patients and that there were no significant differences in demographic factors or medical history between the groups based on nutritional status. The study also highlights the significant correlations between albumin and some laboratory parameters at different assessment stages in both APD and CAPD patients, which may have important implications for the management of these patients. However, due to the scarcity of studies in literature describing metabolic parameters among CAPD and APD patients, we could not compare our results more elaborately. Additional limitations of this study include the relatively small sample size, as only 71 patients underwent APD and 14 patients underwent CAPD, which may limit the generalizability of the findings. Moreover, the unequal sample size of the APD and CAPD groups limited statistical comparison between the groups. Furthermore, the study did not assess the impact of PD modality on survival outcomes, which is an important consideration for patients with ESRD. Overall, it is important to acknowledge these limitations when interpreting the findings of this study.

## Conclusions

The comprehensive evaluation of APD and CAPD patients revealed demographic differences, notably with respect to age and gender distributions. Longitudinal analyses, spanning six months, demonstrated stable laboratory parameters in both patient groups. Furthermore, albumin levels did not significantly correlate with the medical history variables analyzed, such as diabetes or hypertension. However, a consistent and notable relationship was observed between albumin and total protein levels across various months for both patient groups, emphasizing the potential interplay between these parameters in peritoneal dialysis patients. This underscores the importance of regular monitoring of albumin and related parameters for CAPD and APD patients to optimize their clinical management.
